# Supplementary material for: Effect of Nanocrystallization of Anthocyanins Extracted from Two Types of Red-Fleshed Apple Varieties on Its Stability and Antioxidant Activity
Source: Molecules. 2019 Sep 16;24(18):3366. doi: 10.3390/molecules24183366 (PMC6767359; doi:10.3390/molecules24183366)
Supplement: Supplementary file 1 [file molecules-24-03366-s001.pdf]

molecules

Supplementary Materials

Effect of Nanocrystallization of Anthocyanins Extracted from Two Types of Red-Fleshed Apple Varieties on Its Stability and Antioxidant Activity

Jihua Xu [1,2,†], XinXin Li [1,3,†], Shifeng Liu [1,3], Peilei Zhao [1,3], Heqiang Huo [4] and Yugang Zhang [1,3,*]

[1] Qingdao Key Laboratory of Genetic Development and Breeding in Horticultural Plants, Qingdao Agricultural University, Qingdao 266109, China; xujihua@qau.edu.cn (J.X.); lixinxinll@163.com (X.L.); liu18306390438@163.com (S.L.); zhaopeileiyx@163.com (P.Z.)
[2] College of Life Sciences, Qingdao Agricultural University, Qingdao 266109, China
[3] College of Horticulture, Qingdao Agricultural University, Qingdao 266109, China;
[4] Mid-Florida Research and Education Center, University of Florida, Apopka, FL 32703, USA; hhuo@ufl.edu
* Correspondence: ygzhang@qau.edu.cn; Tel.: +86-0532-8608-0752
† These authors contributed equally to this work.

Table S1. Extremely Significant Differential Metabolite Compounds between RL and XJ4.

Metabolite Name	Content		Fold change (XJ4/RL)	VIP
	RL	XJ4		
**Anthocyanins**				
Cyanidin 3-O-malonyl hexoside	9.00E+00	7.80E+05	86666.67	2.41991
Malvidin 3-acetyl-5-diglucoside	8.34E+03	2.43E+05	29.18	1.79473
Peonidin O-hexoside	6.18E+06	8.59E+07	13.91	1.12541
Pelargonidin 3-O-beta-D-glucoside	3.29E+06	4.25E+07	12.92	1.15045
Peonidin 3-O-glucoside chloride	7.62E+06	9.14E+07	12	1.09314
**Flavones**				
Syringetin 5-O-hexoside	9.00E+00	8.19E+05	91037.04	2.42599
Syringetin 7-O-hexoside	9.00E+00	7.53E+05	83629.63	2.41465
Butin	9.00E+00	1.42E+05	15751.85	2.21912
Limocitrin O-hexoside	9.00E+00	1.41E+05	15667	1.56821
C-hexosyl-apigenin O-p-coumaroylhexoside	9.00E+00	7.17E+04	7962.96	2.15034
Tricin 5-O-rutinoside	9.00E+00	4.70E+04	5222.22	2.09581
O-methylChrysoeriol 7-O-hexoside	9.00E+00	3.64E+04	4041.07	1.45597
Selgin O-malonylhexoside	5.44E+04	1.86E+06	34.19	1.18869
Tricin O-saccharic acid	3.07E+04	7.61E+05	24.75	1.2748
Tricin 7-O-hexoside	3.17E+04	6.14E+05	19.37	1.16729
8-C-hexosyl-luteolin O-hexoside	6.77E+03	1.02E+05	15.08	1.62971
C-hexosyl-chrysoeriol O-hexoside	2.66E+04	2.21E+05	8.33	1.66844
Chrysoeriol 8-C-pentosyl-O-rutinoside	5.97E+03	4.72E+04	7.92	1.01184
C-hexosyl-apigenin O-hexosyl-O-hexoside	4.23E+04	3.13E+05	7.39	1.18321
Isovitexin	8.40E+04	1.54E+04	0.18	1.55625
Chrysoeriol 7-O-rutinoside	1.72E+04	2.07E+03	0.12	1.44221
Chrysin C-hexoside	1.49E+05	1.81E+04	0.12	1.63321
Tricin 7-O-acetylglucoside	4.90E+04	9.00E+00	0.01	1.48062
Tricin di-O-hexoside	7.67E+03	9.00E+00	0.01	1.85517
Luteolin 8-C-hexosyl-O-hexoside	7.58E+05	8.66E+04	0.11	1.06046
**Flavonoids**				
Eriocitrin	9.00E+00	4.46E+04	4955.56	2.09402
Schaftoside	9.00E+00	9.21E+03	1023.7	1.88979
Isorhamnetin 3-O-glucoside	2.09E+05	3.08E+06	14.72	1.09312
Persicoside	1.27E+04	1.43E+05	11.27	1.25011
Spinosin	2.34E+04	7.84E+03	0.34	1.37886
Diosmin	3.42E+04	9.00E+00	0.1	2.05246
**Polyphenols**				
Gallocatechin-catechin	9.00E+00	1.54E+04	1711.11	1.95784
6-Gingerol	9.00E+00	1.41E+04	1570.7	1.3703
Catechingallate，CG	9.00E+00	1.22E+04	1355.89	1.36566
**Flavanones**				
Naringeninchalcone	9.00E+00	1.41E+05	15688.89	2.22364
Hesperetin O-malonylhexoside	9.00E+00	9.32E+04	10355.56	2.1862
**Isoflavones**				

Formononetin 7-O-glucoside	5.74E+03	1.39E+05	24.16	1.733
**Flavonols**				
methylQuercetin O-hexoside	1.47E+04	9.57E+05	64.91	1.93511
Isorhamnetin O-acetyl-hexoside	6.68E+05	2.87E+07	43.02	1.41281
Myricetin 3-O-galactoside	1.42E+05	4.00E+06	28.12	1.30523
Isorhamnetin O-hexoside	1.51E+06	2.17E+07	14.37	1.08324
Isorhamnetin 5-O-hexoside	1.50E+06	2.04E+07	13.61	1.06
Myricetin 3-O-rhamnoside (Myricitrin)	9.15E+05	1.22E+07	13.27	1.146
Syringetin 3-O-hexoside	3.29E+04	1.40E+05	4.27	1.05705
Quercetin 7-O-rutinoside	5.51E+06	5.86E+05	0.11	1.0872
Quercetin 3-O-rutinoside (Rutin)	1.95E+06	2.11E+05	0.11	1.07078

Note: VIP means variable importance in projection; VIP > 1 and fold change > 2 (upregulation) or fold change < 0.5 (down regulation) were set as the cutoff for extremely significant differential metabolite compounds.

© 2019 by the authors. Licensee MDPI, Basel, Switzerland. This article is an open access article distributed under the terms and conditions of the Creative Commons Attribution (CC BY) license (http://creativecommons.org/licenses/by/4.0/).
